# 3-Methyl­piperidinium bromide

**DOI:** 10.1107/S160053681201971X

**Published:** 2012-05-05

**Authors:** Qian Xu

**Affiliations:** a Ordered Matter Science Research Center, Southeast University, Nanjing 211189, People’s Republic of China

## Abstract

In the crystal structure of the title molecular salt, C_6_H_14_N^+^·Br^−^, N—H⋯Br hydrogen bonds link the cations and anions to form a one-dimensional network.

## Related literature

For general background to ferroelectric organic frameworks, see: Ye *et al.* (2006 ▶); Zhang *et al.* (2008 ▶, 2010 ▶).
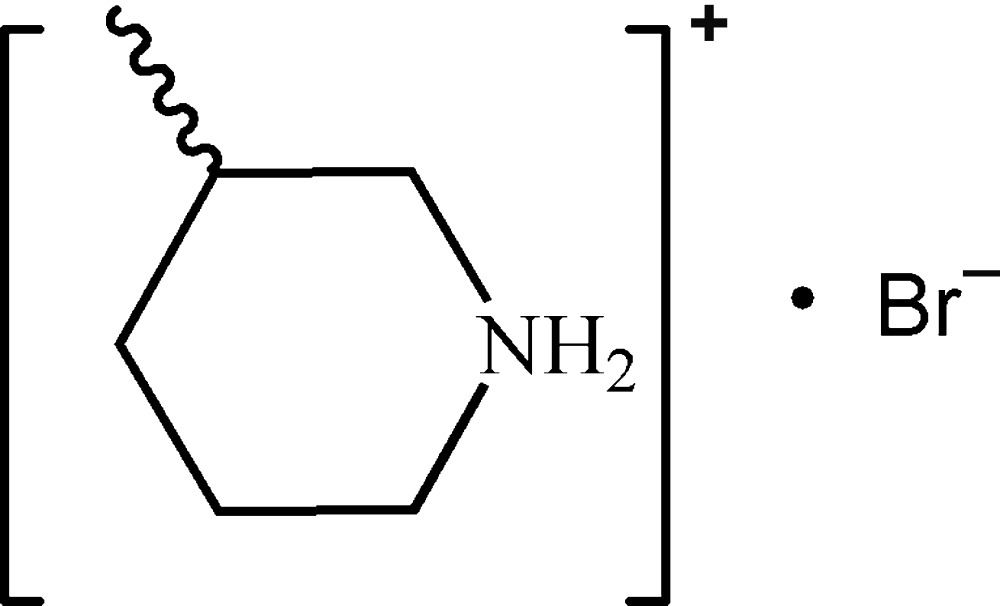


## Experimental

### 

#### Crystal data

C_6_H_14_N^+^·Br^−^
*M* *_r_* = 180.09
Monoclinic, 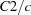

*a* = 23.134 (5) Å
*b* = 9.997 (2) Å
*c* = 7.7214 (15) Å
β = 107.90 (3)°
*V* = 1699.3 (6) Å^3^
*Z* = 8
Mo *K*α radiation
μ = 4.75 mm^−1^
*T* = 293 K
0.55 × 0.44 × 0.36 mm

#### Data collection

Rigaku Mercury70 CCD diffractometer
Absorption correction: multi-scan (*CrystalClear*; Rigaku, 2005 ▶) *T*_min_ = 0.134, *T*_max_ = 0.223
8544 measured reflections
1946 independent reflections
1327 reflections with *I* > 2σ(*I*)
*R*_int_ = 0.057

#### Refinement

*R*[*F* ^2^ > 2σ(*F* ^2^)] = 0.043
*wR*(*F* ^2^) = 0.091
*S* = 1.09
1946 reflections
74 parameters
H-atom parameters constrained
Δρ_max_ = 0.37 e Å^−3^
Δρ_min_ = −0.57 e Å^−3^

### 

Data collection: *SCXmini* (Rigaku, 2006 ▶); cell refinement: *SCXmini*; data reduction: *SCXmini*; program(s) used to solve structure: *SHELXS97* (Sheldrick, 2008 ▶); program(s) used to refine structure: *SHELXL97* (Sheldrick, 2008 ▶); molecular graphics: *DIAMOND* (Brandenburg & Putz, 2005 ▶); software used to prepare material for publication: *SHELXL97*.

## Supplementary Material

Crystal structure: contains datablock(s) I, global. DOI: 10.1107/S160053681201971X/ds2192sup1.cif

Structure factors: contains datablock(s) I. DOI: 10.1107/S160053681201971X/ds2192Isup2.hkl

Supplementary material file. DOI: 10.1107/S160053681201971X/ds2192Isup3.cml

Additional supplementary materials:  crystallographic information; 3D view; checkCIF report

## Figures and Tables

**Table 1:** Hydrogen-bond geometry (Å, °)

*D*—H⋯*A*	*D*—H	H⋯*A*	*D*⋯*A*	*D*—H⋯*A*
N1—H1*D*⋯Br1	0.90	2.38	3.273 (3)	175
N1—H1*C*⋯Br1^i^	0.90	2.36	3.255 (3)	173

Symmetry code: (i) 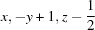.

**Fig. 1.**
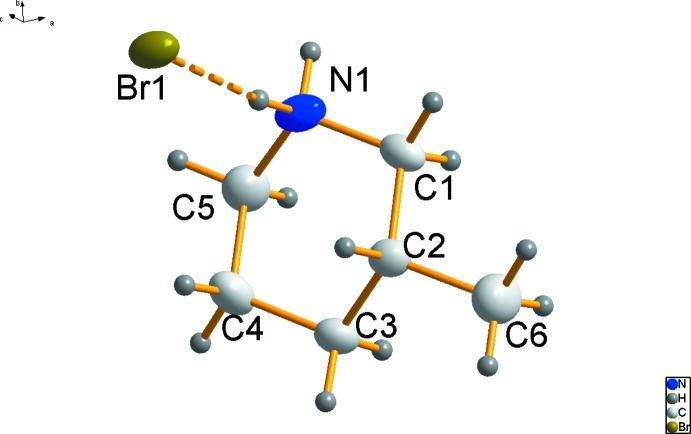
The molecular structure of the title compound, with the atomic numbering scheme. Displacement ellipsoids are drawn at the 30% probability level. The dashed line indicate intramolecular hydrogen bond.

**Fig. 2.**
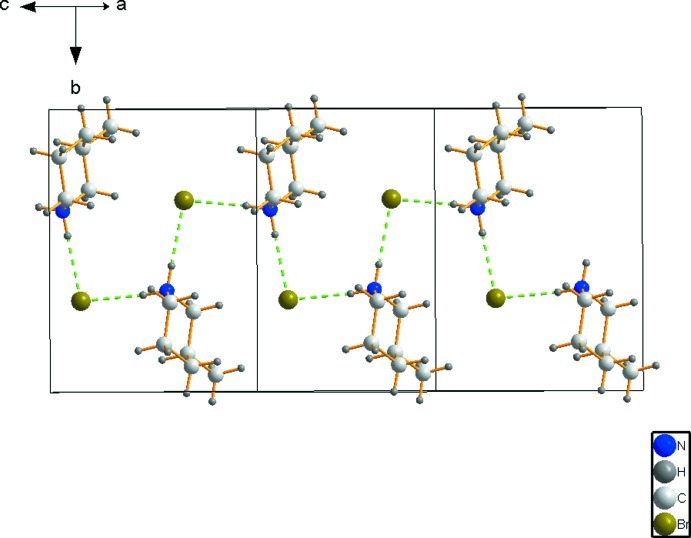
A view of the packing of the title compound, stacking along the a axis. Dashed lines indicate hydrogen bonds.

## supplementary crystallographic information

### Comment

Dielectric-ferroelectric constitute an interesting class of materials, comprising organic ligands,metal-organic coordination compounds and organic-inorganic hybrids.(Zhang *et al.*, 2010; Zhang *et al.*, 2008;Ye *et al.*, 2006). Unfortunately,the dielectric constant of the title compound as a function of temperature indicates that the permittivity is basically temperature-independent, below the melting point (428k-429k) of the compound, we have found that title compound has no dielectric disuniform from 80 K to 405 K. Herein we descibe the crystal structure of this compound.

Regarding its crystal structure,the asymmetric unit of the title compound consists of a 3-methylpiperidinium cation, a bromide anion (Fig. 1). The cations and anions were connected by hydrogen bonds involving N—H···Br which makes great contribution to the stability of the crystal structure,and these hydrogen bonds link the cations and anions into stable crystal structure (Fig. 2 and Tab. 1).

### Experimental

The title compound was obtained by the addition of hydrobromic acid (0.8 g, 0.01 mol) to a solution of 3-methylpiperidine (0.97 g, 0.01 mol) in water, in the stoichiometric ratio 1: 1. Good quality single crystals were obtained by slow evaporation after two days(the chemical yield is 65%).

### Refinement

Amino H atoms were located in a difference Fourier map and refined isotropically. Other H atoms were placed in geometrically idealized positions nd constrained to ride on their parent atoms with C—H = 0.97–0.98 Å, *U*_iso_(H) = 1.2*U*_iso_(C, N) and *U*_iso_(H) = 1.5*U*_iso_(C) for the methyl.

### Crystal data

**Table:**

C_6_H_14_N^+^·Br^−^	*F*(000) = 736
*M**_r_* = 180.09	*D*_x_ = 1.408 Mg m^−^^3^
Monoclinic, *C*2/*c*	Mo *K*α radiation, λ = 0.71073 Å
Hall symbol: -C 2yc	Cell parameters from 1946 reflections
*a* = 23.134 (5) Å	θ = 3.0–27.5°
*b* = 9.997 (2) Å	µ = 4.75 mm^−^^1^
*c* = 7.7214 (15) Å	*T* = 293 K
β = 107.90 (3)°	Block, colorless
*V* = 1699.3 (6) Å^3^	0.55 × 0.44 × 0.36 mm
*Z* = 8	

### Data collection

**Table:**

Rigaku Mercury70 CCD diffractometer	1946 independent reflections
Radiation source: fine-focus sealed tube	1327 reflections with *I* > 2σ(*I*)
Graphite monochromator	*R*_int_ = 0.057
ω scans	θ_max_ = 27.5°, θ_min_ = 3.3°
Absorption correction: multi-scan (*CrystalClear*; Rigaku, 2005)	*h* = −30→30
*T*_min_ = 0.134, *T*_max_ = 0.223	*k* = −12→12
8544 measured reflections	*l* = −10→10

### Refinement

**Table:**

Refinement on *F*^2^	Secondary atom site location: difference Fourier map
Least-squares matrix: full	Hydrogen site location: inferred from neighbouring sites
*R*[*F*^2^ > 2σ(*F*^2^)] = 0.043	H-atom parameters constrained
*wR*(*F*^2^) = 0.091	*w* = 1/[σ^2^(*F*_o_^2^) + (0.035*P*)^2^ + 0.5231*P*] where *P* = (*F*_o_^2^ + 2*F*_c_^2^)/3
*S* = 1.09	(Δ/σ)_max_ < 0.001
1946 reflections	Δρ_max_ = 0.37 e Å^−^^3^
74 parameters	Δρ_min_ = −0.57 e Å^−^^3^
0 restraints	Extinction correction: *SHELXL97* (Sheldrick, 2008)
Primary atom site location: structure-invariant direct methods	Extinction coefficient: 0

### Special details

**Table:**

Geometry. All e.s.d.'s (except the e.s.d. in the dihedral angle between two l.s. planes) are estimated using the full covariance matrix. The cell e.s.d.'s are taken into account individually in the estimation of e.s.d.'s in distances, angles and torsion angles; correlations between e.s.d.'s in cell parameters are only used when they are defined by crystal symmetry. An approximate (isotropic) treatment of cell e.s.d.'s is used for estimating e.s.d.'s involving l.s. planes.
Refinement. Refinement of *F*^2^ against ALL reflections. The weighted *R*-factor *wR* and goodness of fit *S* are based on *F*^2^, conventional *R*-factors *R* are based on *F*, with *F* set to zero for negative *F*^2^. The threshold expression of *F*^2^ > σ(*F*^2^) is used only for calculating *R*-factors(gt) *etc*. and is not relevant to the choice of reflections for refinement. *R*-factors based on *F*^2^ are statistically about twice as large as those based on *F*, and *R*- factors based on ALL data will be even larger.

### Fractional atomic coordinates and isotropic or equivalent isotropic displacement parameters (Å^2^)

**Table:**

	*x*	*y*	*z*	*U*_iso_*/*U*_eq_	
N1	0.11379 (15)	0.3573 (3)	0.1464 (4)	0.0570 (9)	
H1C	0.1188	0.4456	0.1332	0.068*	
H1D	0.1141	0.3431	0.2618	0.068*	
C1	0.16511 (17)	0.2840 (4)	0.1151 (5)	0.0513 (9)	
H1A	0.2027	0.3117	0.2043	0.062*	
H1B	0.1676	0.3057	−0.0048	0.062*	
C2	0.15735 (16)	0.1344 (3)	0.1290 (5)	0.0468 (9)	
H2	0.1575	0.1135	0.2531	0.056*	
C3	0.09709 (15)	0.0903 (4)	−0.0014 (5)	0.0523 (9)	
H3A	0.0916	−0.0047	0.0134	0.063*	
H3B	0.0969	0.1057	−0.1256	0.063*	
C4	0.04502 (18)	0.1672 (4)	0.0339 (6)	0.0634 (11)	
H4A	0.0070	0.1406	−0.0543	0.076*	
H4B	0.0430	0.1456	0.1543	0.076*	
C5	0.05371 (18)	0.3159 (4)	0.0204 (6)	0.0641 (11)	
H5A	0.0514	0.3390	−0.1036	0.077*	
H5B	0.0216	0.3632	0.0511	0.077*	
C6	0.21027 (17)	0.0600 (4)	0.0926 (6)	0.0775 (13)	
H6A	0.2043	−0.0346	0.0993	0.116*	
H6B	0.2476	0.0854	0.1821	0.116*	
H6C	0.2120	0.0827	−0.0265	0.116*	
Br1	0.120008 (18)	0.32311 (4)	0.57328 (5)	0.05608 (17)	

### Atomic displacement parameters (Å^2^)

**Table:**

	*U*^11^	*U*^22^	*U*^33^	*U*^12^	*U*^13^	*U*^23^
N1	0.091 (3)	0.0367 (17)	0.0462 (18)	−0.0022 (16)	0.0255 (17)	0.0003 (15)
C1	0.054 (2)	0.049 (2)	0.047 (2)	−0.0167 (18)	0.0104 (18)	−0.0017 (18)
C2	0.053 (2)	0.0416 (19)	0.045 (2)	−0.0015 (17)	0.0131 (17)	0.0016 (17)
C3	0.053 (2)	0.040 (2)	0.061 (2)	−0.0077 (17)	0.0138 (19)	−0.0038 (19)
C4	0.052 (2)	0.053 (2)	0.085 (3)	−0.0089 (19)	0.020 (2)	0.004 (2)
C5	0.061 (3)	0.055 (2)	0.074 (3)	0.007 (2)	0.017 (2)	0.006 (2)
C6	0.059 (3)	0.080 (3)	0.090 (3)	0.002 (2)	0.017 (2)	−0.009 (3)
Br1	0.0834 (3)	0.0399 (2)	0.0459 (2)	−0.00318 (19)	0.0213 (2)	−0.00200 (18)

### Geometric parameters (Å, º)

**Table:**

N1—C1	1.477 (5)	C3—H3A	0.9700
N1—C5	1.490 (5)	C3—H3B	0.9700
N1—H1C	0.9000	C4—C5	1.507 (5)
N1—H1D	0.9000	C4—H4A	0.9700
C1—C2	1.514 (5)	C4—H4B	0.9700
C1—H1A	0.9700	C5—H5A	0.9700
C1—H1B	0.9700	C5—H5B	0.9700
C2—C3	1.512 (5)	C6—H6A	0.9600
C2—C6	1.530 (5)	C6—H6B	0.9600
C2—H2	0.9800	C6—H6C	0.9600
C3—C4	1.523 (5)		
			
C1—N1—C5	113.0 (3)	C2—C3—H3B	109.5
C1—N1—H1C	109.3	C4—C3—H3B	109.5
C5—N1—H1C	109.0	H3A—C3—H3B	108.1
C1—N1—H1D	108.7	C5—C4—C3	110.8 (3)
C5—N1—H1D	109.0	C5—C4—H4A	109.5
H1C—N1—H1D	107.8	C3—C4—H4A	109.5
N1—C1—C2	111.1 (3)	C5—C4—H4B	109.5
N1—C1—H1A	109.4	C3—C4—H4B	109.5
C2—C1—H1A	109.4	H4A—C4—H4B	108.1
N1—C1—H1B	109.4	N1—C5—C4	110.3 (3)
C2—C1—H1B	109.4	N1—C5—H5A	109.6
H1A—C1—H1B	108.0	C4—C5—H5A	109.6
C3—C2—C1	110.2 (3)	N1—C5—H5B	109.6
C3—C2—C6	111.2 (3)	C4—C5—H5B	109.6
C1—C2—C6	110.4 (3)	H5A—C5—H5B	108.1
C3—C2—H2	108.3	C2—C6—H6A	109.5
C1—C2—H2	108.3	C2—C6—H6B	109.5
C6—C2—H2	108.3	H6A—C6—H6B	109.5
C2—C3—C4	110.6 (3)	C2—C6—H6C	109.5
C2—C3—H3A	109.5	H6A—C6—H6C	109.5
C4—C3—H3A	109.5	H6B—C6—H6C	109.5
			
C5—N1—C1—C2	−56.3 (4)	C6—C2—C3—C4	−178.8 (3)
N1—C1—C2—C3	55.7 (4)	C2—C3—C4—C5	56.6 (5)
N1—C1—C2—C6	178.9 (3)	C1—N1—C5—C4	56.1 (4)
C1—C2—C3—C4	−56.1 (4)	C3—C4—C5—N1	−55.5 (5)

### Hydrogen-bond geometry (Å, º)

**Table:**

*D*—H···*A*	*D*—H	H···*A*	*D*···*A*	*D*—H···*A*
N1—H1*D*···Br1	0.90	2.38	3.273 (3)	175
N1—H1*C*···Br1^i^	0.90	2.36	3.255 (3)	173

Symmetry code: (i) *x*, −*y*+1, *z*−1/2.
